# Supplementary material for: Population-level antagonism between FGF and BMP signaling steers mesoderm differentiation in embryonic stem cells
Source: Biol Open. 2023 Aug 17;12(8):bio059941. doi: 10.1242/bio.059941 (PMC10445724; doi:10.1242/bio.059941)
Supplement: Supplementary information [file biolopen-12-059941-s1.pdf]

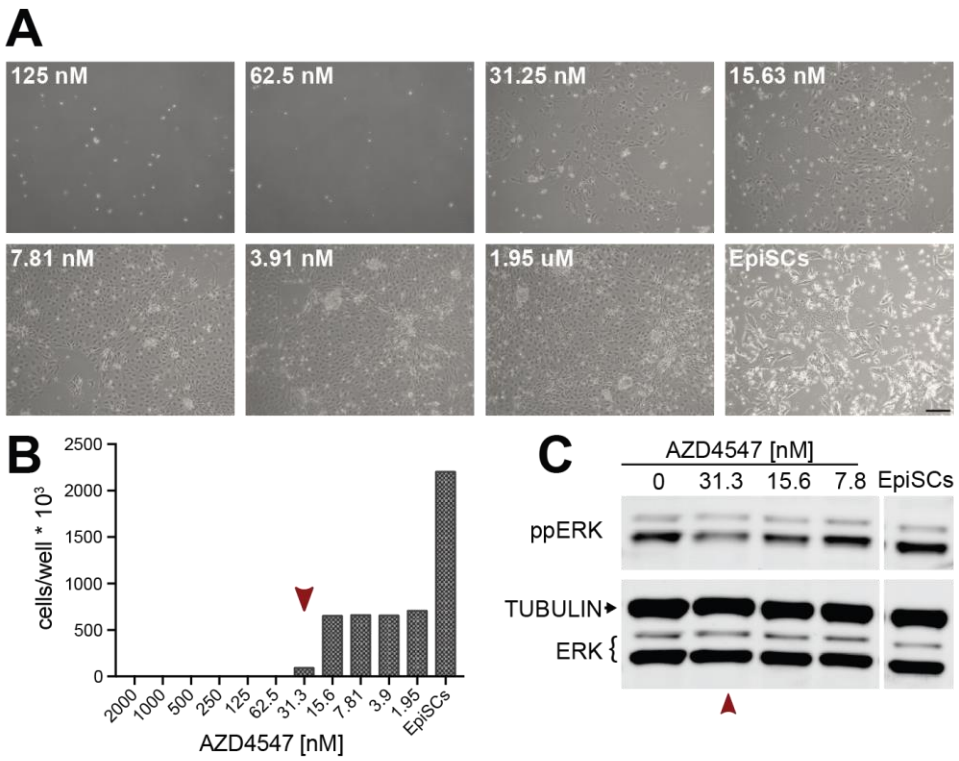

Fig. S1. Titration of the FGFR inhibitor AZD4547 during mesoderm differentiation. A Brightfield images of cells after differentiation for 3 days with 1 µM Chi, 8 ng/ml BMP4 and indicated concentrations of AZD4547. Scale bar, 250 µm. **B** Cell count after treatment as in **A**. **C** Immunoblotting for ppERK and total ERK of cells treated for 24 h with 1 µM Chiron, 8 ng/ml BMP4 and indicated concentrations of AZD4547. Red arrow in **B**, **C** indicates highest AZD concentration compatible with cell survival in this experiment.

Biology Open • Supplementary information

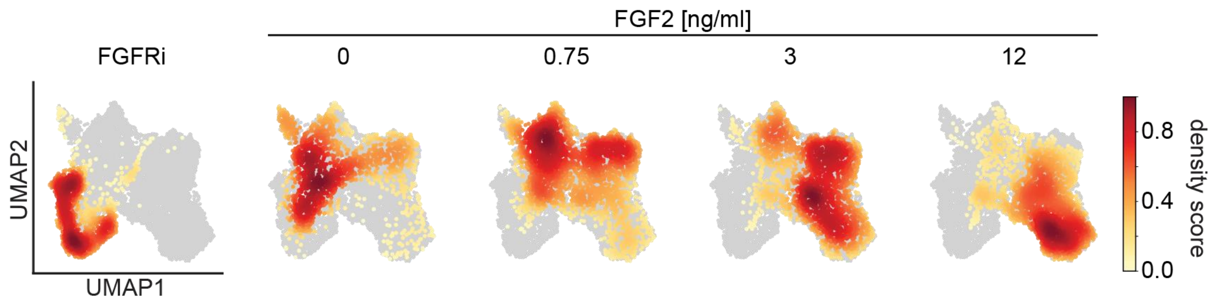

Fig. S2. Distribution of cells from individual samples in UMAP space. Density plots showing the distribution of cells from each sample in the UMAP plot from Fig. 3D. Remaining cells from the dataset are shown in gray.

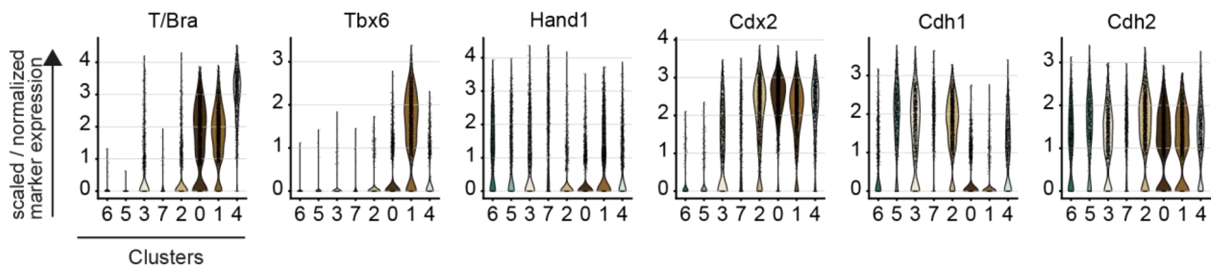

Fig. S3. Expression of marker genes in cell clusters identified by scRNAseq. Violin plots showing the expression levels of *T/Bra*, *Tbx6*, *Hand1*, *Cdx2*, *Cdh1* and *Cdh2* for clusters identified in Fig. 3E. The width of the violin plots is scaled per number of observations.

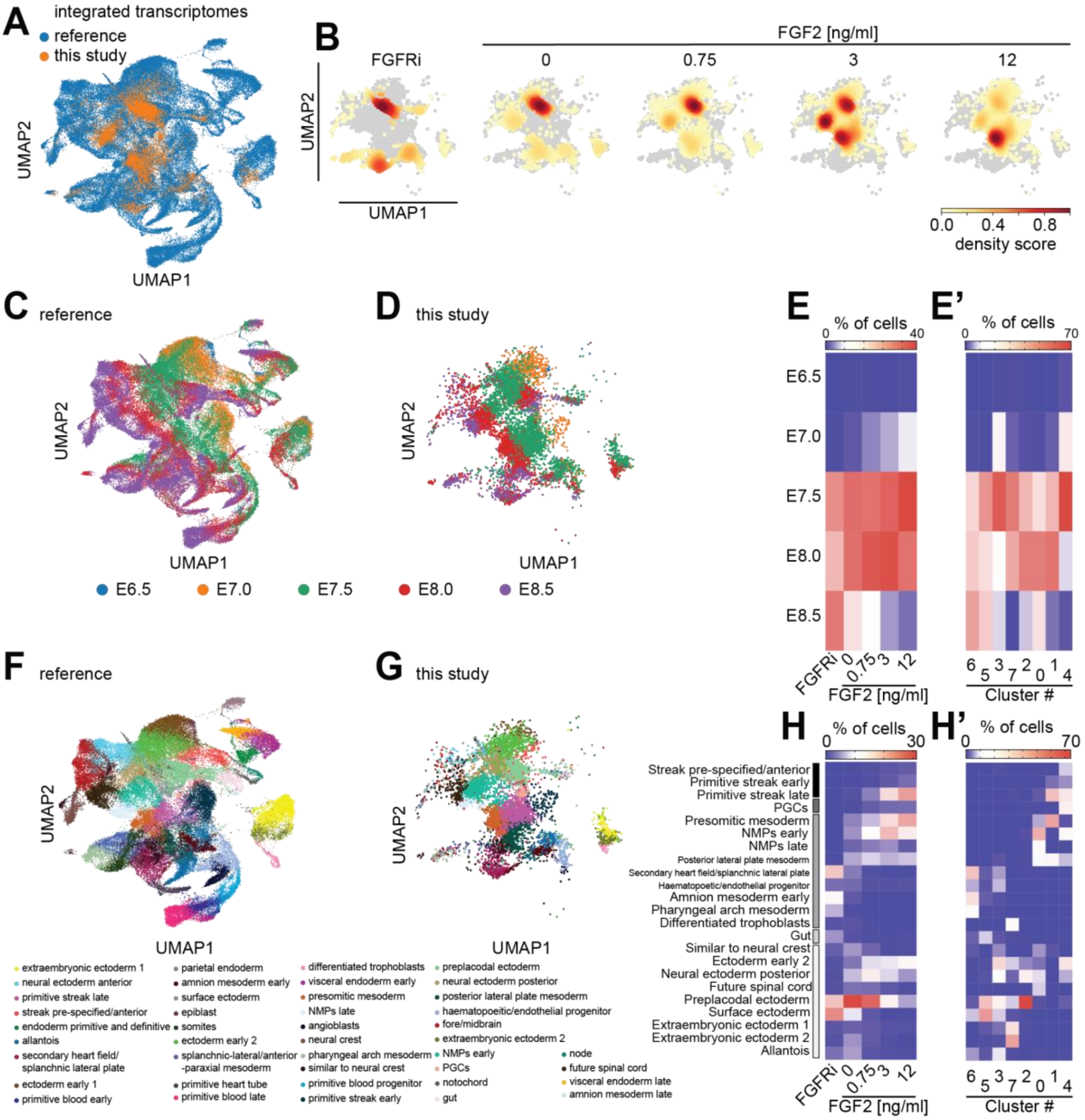

Fig. S4. Integration of single cell transcriptomes with an alternative embryo reference reveals developmental stages and cell types obtained in vitro. A UMAP representation of single cell transcriptomes from the reference dataset by Grosswendt et al., 2020 (bue) and the in vitro dataset (orange) after asymmetric integration. **B** Transcriptomes from each of the FGF treatment regimes shown separately as density plots, compared to transcriptomes of all other in vitro differentiated cells shown in gray. **C - E'** Label transfer to identify developmental stages represented in vitro. **C** UMAP plot of reference dataset with color-coding according to developmental stage of cells. **D** UMAP representation of in vitro differentiated cells after integration, color-coded according to stage label transferred from the reference dataset. **E** Heatmap showing proportion of cells that were

Biology Open • Supplementary information

assigned a specific stage label for different FGF signaling strengths. **E'** Same as **E**, but showing proportion of cells from each of the clusters identified in Fig. 3E that were assigned a specific stage label. **F - H'** Label transfer to identify cell types represented in vitro. **F** UMAP plot of reference dataset with color-coding according to cell identity. **G** Same display of transcriptomes from in vitro differentiated cells as in **D**, but color-coded according to cell-type label transferred from the reference dataset. **H** Heatmap showing proportion of cells that were assigned a specific cell type label for different FGF signaling strengths. **H'** Same as **E'**, but showing proportion of cells from each of the clusters identified in Fig. 3E that were assigned a specific stage label. Cell types in **E**, **E'**, **H**, **H'** are ordered from top to bottom: pluripotent epiblast-like cell types, primordial germ cells (PGCs), mesoderm subtypes, endoderm, and ectoderm-related cell types.

Biology Open • Supplementary information

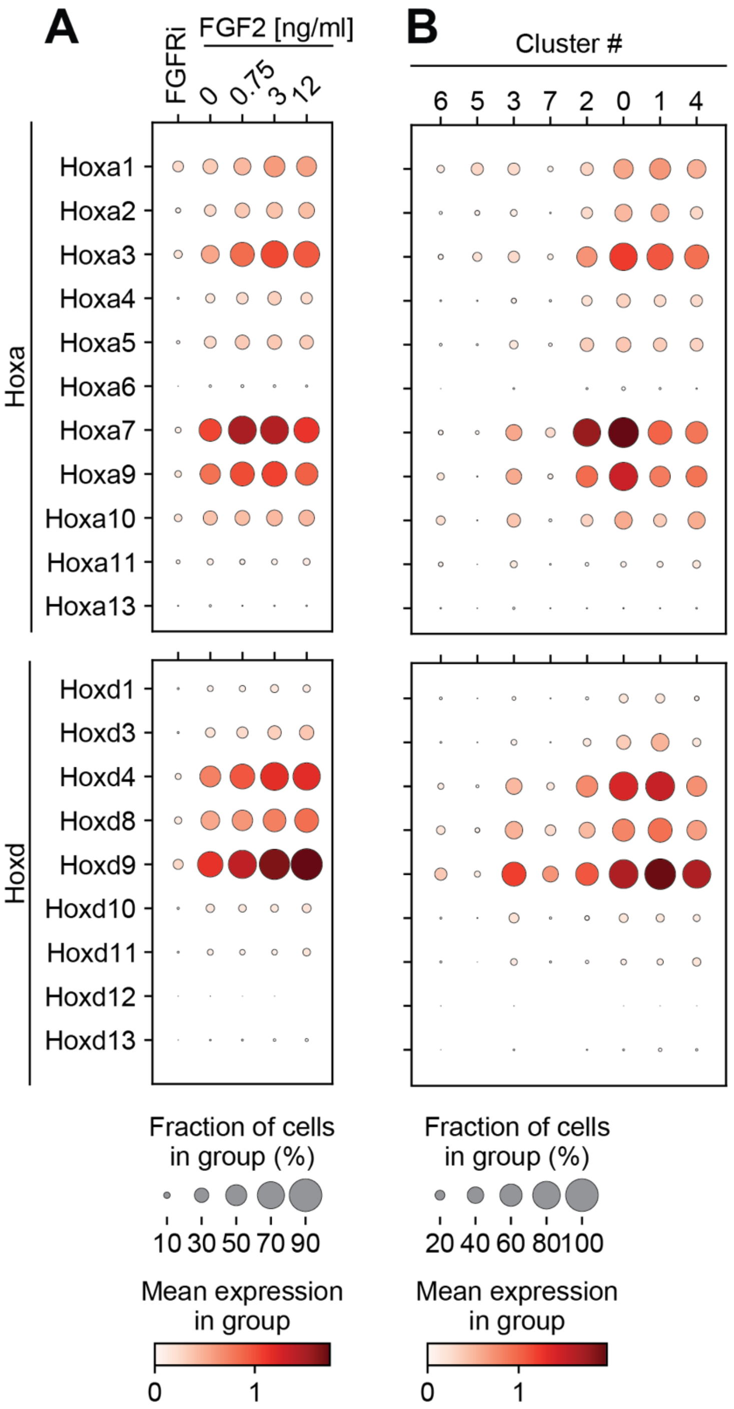

Fig. S5. FGF- and cluster-specific expression of Hox genes. A Dot plot showing expression of Hoxa (top) and Hoxd genes (bottom) depending of FGF signaling levels. Size of the dot indicates the fraction of cells in each group in which expression of a specific gene was detected, color indicates mean expression level. **B** Same as **A**, but now showing Hox gene expression in the clusters identified in Figure 3E.

Biology Open • Supplementary information

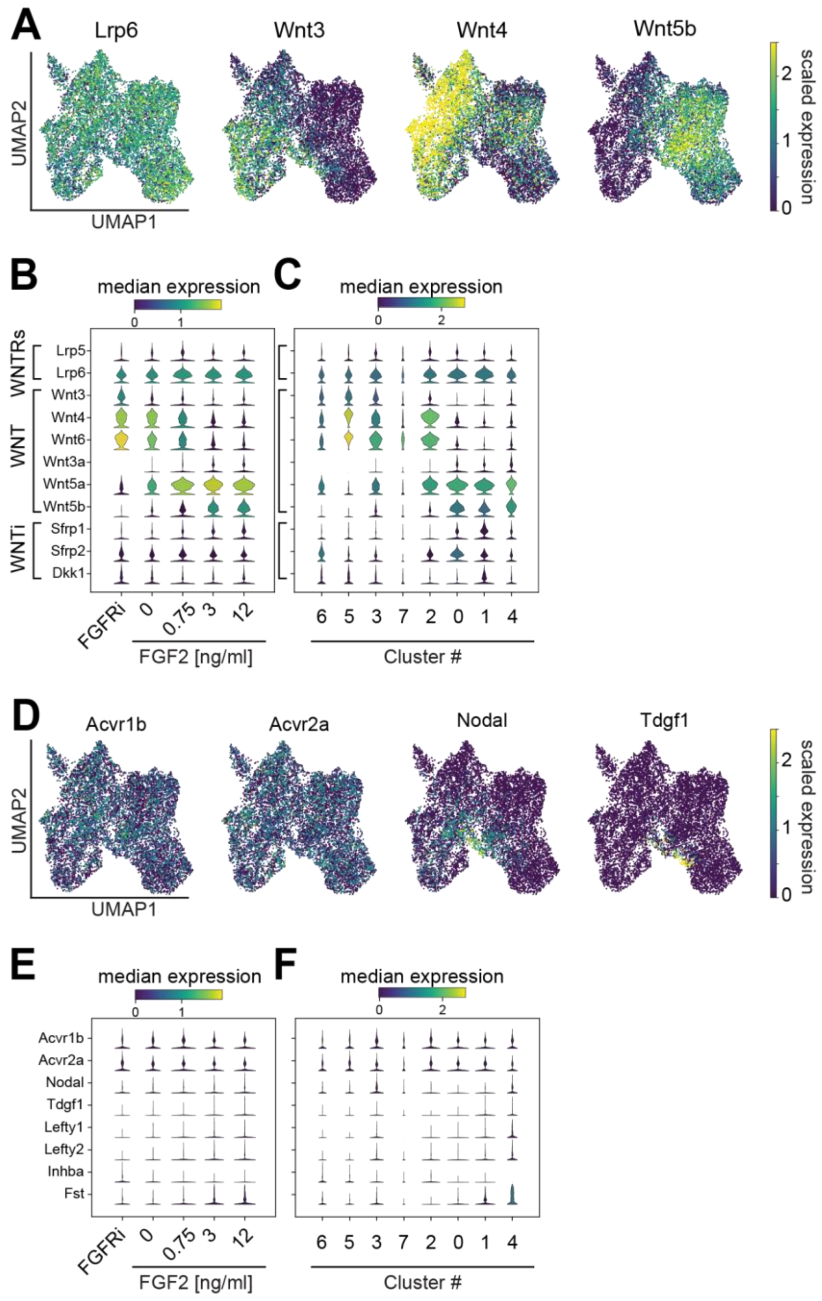

Fig. S6. FGF- and cluster-specific expression of Wnt and Nodal signaling genes. **A** Single-cell expression of the Wnt receptor *Lrp6* and ligands *Wnt3*, *Wnt4*, and *Wnt5b*. Single cells are represented on the same UMAP plot as in Fig. 3D, expression levels of the individual genes are color-coded. **B** Stacked violin plots showing the expression of a panel of genes associated with Wnt signaling for each of the FGF signaling regimes. **C** Expression levels of same genes as in **B**, but segregated according to clusters identified in Fig. 3E. **D** Same as **A**, but for the Nodal receptors *Acvr1b* and *Acvr2a*, and the ligands *Nodal* and *Tdgf1*. **E**, **F** Same as **B**, **C**, but for a panel of genes associated with Activin/Nodal signaling. Width of the violin plots in **B**, **C**, **E**, **F** indicates the number of observations, their color reflects the median expression of the selected gene for the cells contained in each observation.

Biology Open • Supplementary information

Table S1. Summary statistics of single cell sequencing dataset.

Sample	number of cells	average number of genes	average number of reads	% reads mapped to mitochondrial genes
**all**	10653	5294	30185	4.3
**FGFRi**	1701	5088	28116	4.5
**0 ng/ml FGF2**	1696	5472	33498	4.3
**0.75 ng/ml FGF2**	2725	5277	29923	4.1
**3 ng/ml FGF2**	2125	5362	30403	4.3
**12 ng/ml FGF2**	2406	5273	29417	4.3

Biology Open • Supplementary information

Table S2. Table shows number of cells from every experimental condition that was allocated to each of the eight clusters identified in Fig. 3E.

	Cluster							
**Sample**	**0**	**1**	**2**	**3**	**4**	**5**	**6**	**7**
**FGFRi**	17	0	23	111	1	757	758	34
**0 ng/ml FGF2**	334	110	435	515	14	116	52	120
**0.75 ng/ml FGF2**	778	320	851	429	228	18	3	98
**3 ng/ml FGF2**	661	557	359	146	378	0	0	24
**12 ng/ml FGF2**	453	1089	201	218	435	0	0	10

Biology Open • Supplementary information

Table S3. Sequences of primers used in RT-qPCR experiments.

Gene	Forward primer	Reverse primer	efficiency
Actin	GCAGGAGTACGATGAGTCCG	ACGCAGCTCAGTAACAGTCC	2.00
Tbp	GTGCCAGATACATTCCGCCT	CAAGCTGCGTTTTTGTGCA	2.00
T/Bra	CTGGGAGCTCAGTTCTTTCG	GTCCACGAGGCTATGAGGAG	1.94
Hand1	GAGGAGAGGAAAGGACGCAG	CTCGGCGGGAAGTGAACATA	2.00
Gata6	TGGGAGCCATTTGGTCTATC	GACCTCAGATCAGCCACGTT	2.00
Tbx6	GGCAGCTCCATCTGTACCAT	ACCGAGGCTCAGTACATTGG	1.92
Msgn1	CCAGAAAGGCAGCAAGTCA	GAGGAGGTCTGTGAGTTCCC	2.00
Foxa2	CATTACGCCTTCAACCACCC	GGTAGTGCATGACCTGTTCG	1.92
Shh	AAAGCTGACCCCTTTAGCCTA	TTCGGAGTTTCTTGTGATCTTCC	2.00
